# Pericarditis as a trigger for Prinzmetal angina – a case report

**DOI:** 10.25122/jml-2021-0061

**Published:** 2021

**Authors:** Hossein Sheibani, Mojgan Javedani Masroor

**Affiliations:** 1. Clinical Research Development Unit, Imam Hossein Hospital, Shahroud University of Medical Science, Shahroud, Iran; 2. Shahid Akbar-Abadi Clinical Research Development Unit, Iran University of Medical Science, Tehran, Iran

**Keywords:** Pericarditis, prinzmetal angina, CCU – Critical Cardiac Unit, ECG – Electrocardiogram, RV – Right Ventricle, MI – Myocardial Infarction, NSAID – Non-Steroidal Anti-Inflammatory Drugs, TNG – Trinitroglycerin

## Abstract

Prinzmetal angina is one of the causes of acute coronary syndromes, the exact etiology of which is still unknown. Here we introduce a 27-year-old man with no history of cardiovascular disease, with a history of hospitalization due to acute pericarditis in the previous month, who was discharged with a good response to ibuprofen treatment but had clinical and electrocardiographically recurrence of pericarditis with compressive retrosternal chest pain and electrocardiogram (ECG) changes in favor of acute infero-postero-right ventricular (RV) myocardial infarction (MI). Treatment with vasodilator improved compressive retrosternal chest pain and reversed acute myocardial infarction changes completely and left pleuritic chest pain and pericarditis changes in the ECG. Due to the typical chest pain, he was admitted to the emergency room; ECG revealed generalized ST-segment elevation with acute pericarditis pattern again. Acute infero-posterior and right ventricular acute myocardial infarction pattern was also evident. After treatment with nitroglycerin in the Critical Cardiac Unit (CCU), all ECG ischemic changes returned to baseline, and pericarditis remained in all leads. The patient was discharged with non-steroidal anti-inflammatory drugs (NSAIDs), calcium channel blockers, and a good general condition.

## Introduction

Acute coronary syndromes are one of the most common causes of hospitalization in the Critical Cardiac Unit (CCU), which in the vast majority of cases is due to significant stenosis (more than 50%) in the lumen of the coronary artery in angiography [1]. The most important underlying factor in all forms of ischemic heart disease is atherosclerosis, rooted in the lifestyle and mediation of risk factors such as smoking, diabetes, high blood pressure, inactivity etc. However, in 1–14% of cases, an acute myocardial infarction occurs without coronary artery stenosis [2, 3]. In prinzmetal angina, the pathogenesis of the disease is coronary artery spasm. However, the causes and risk factors are not well known. Prinzmetal angina is usually considered equivalent to spasmodic angina and can be a cause of myocardial infarction [4].

Coronary artery spasm is an intense vasoconstriction of the coronary arteries and may be responsible for myocardial ischemia, myocardial infarction, and sudden deaths. Coronary angiography is generally needed to identify the cause. Coronary artery spasm is a multifactorial disease with an underlying mechanism still poorly understood [5]. Coronary vasospasm has been reported to coexist with myocarditis, possibly due to endothelial dysfunction or coronary smooth muscle cell hyperreactivity [6].

We do not yet know what causes vasospastic angina. However, we do know that the following are often triggers: emotional stress, exposure to extreme cold weather or a sudden drop in temperature, hyperventilation, allergic reactions (usually severe reactions that result in histamine release, sometimes referred to as Kounis Syndrome), inflammation of the coronary artery wall, smoking, some antidepressants, some anti-migraine drugs, use of cocaine, high consumption of alcohol [6]. Angina pain in vasospastic angina manifests as pain or discomfort in the chest, arm, or jaw. The pain is often severe but can be variable and usually occurs while at rest and early in the morning or late at night [6].

## Case Report

**Figure 1.**
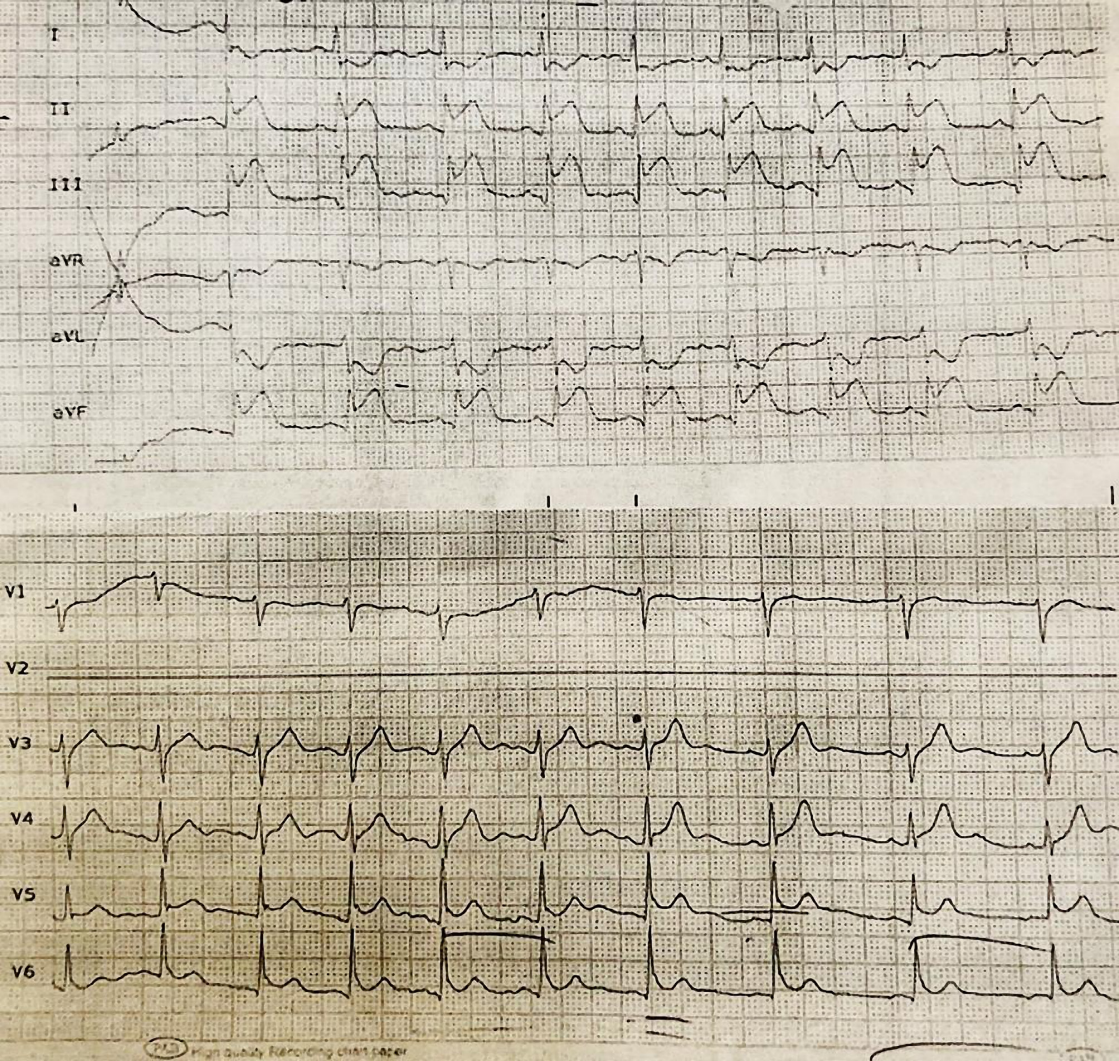
The first electrocardiogram in the emergency room demonstrated two types of ST-segment elevation in inferior and precordial leads in favor of acute MI and acute pericarditis, respectively.

**Figure 2.**
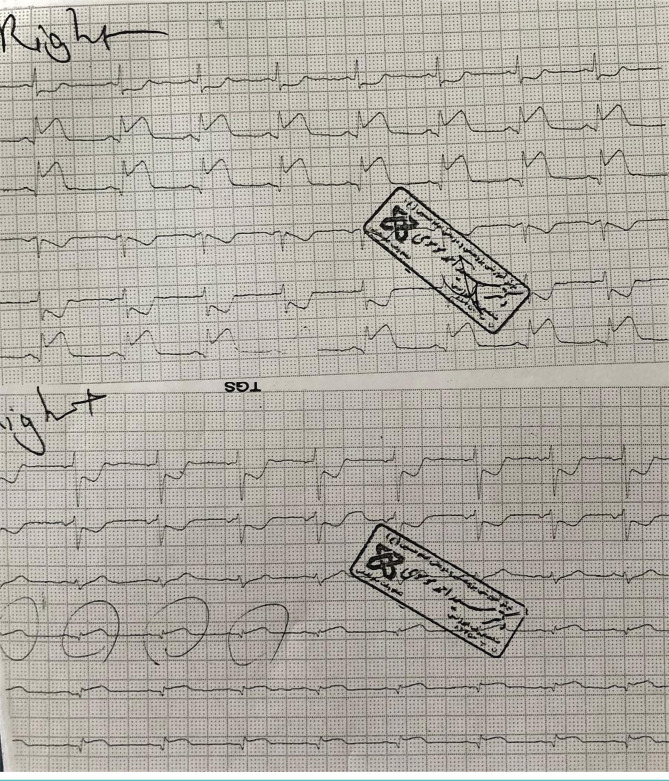
Posterior and right-sided ECG leads in the first electrocardiogram from the emergency room that demonstrated ST-segment elevation in posterior and right ventricle leads.

The patient was a 28 years old man, an ordinary worker without any remarkable history and cardiovascular risk factors, who was admitted with a complaint of retrosternal chest pain and heaviness one hour before hospitalization in the emergency room. The first electrocardiogram demonstrated localized ST-segment elevation with a pattern in favor of acute infero postero-right ventricular (RV) myocardial infarction, accompanied by generalized ST-segment elevation in precordial leads in favor of acute pericarditis (Figures 1, 2).

**Figure 3.**
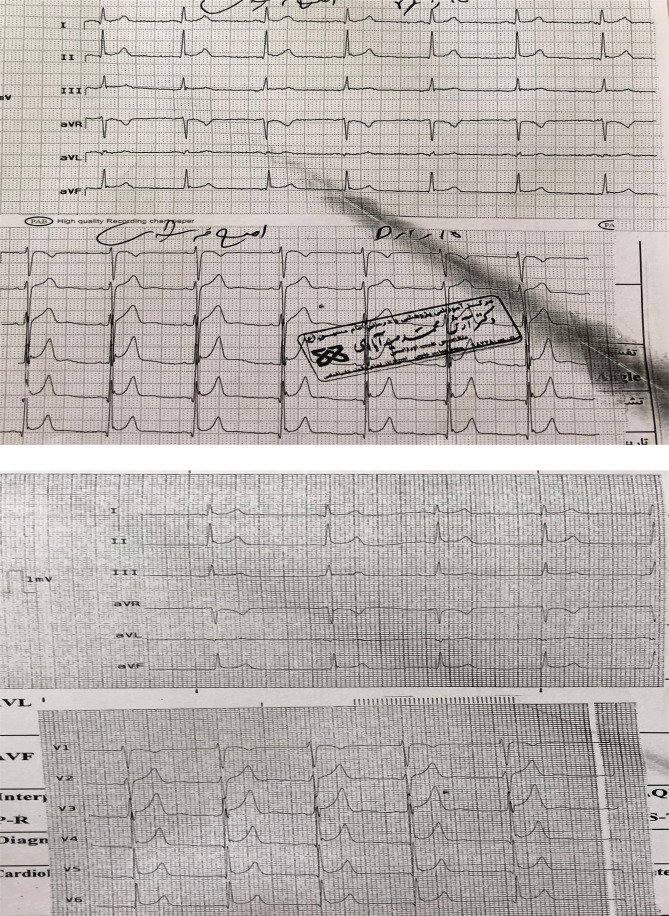
Electrocardiogram prior to patient's hospitalization two months ago due to acute pericarditis; acute phase of patient's pericarditis that demonstrated generalized ST-segment elevation.

After verifying the patient’s medical history, we identified that the patient was hospitalized two months ago, with acute pericarditis, generalized ST-elevation, and normal transthoracic echocardiography, and received treatment for acute pericarditis. Following a good response to treatment, the patient was discharged in good general condition with ibuprofen (Figure 3).

The patient continued to take ibuprofen for 10 days after discharge. Twenty-four hours prior to this hospitalization, he suffered from pleuritic chest pain similar to the pain of the previous hospitalization and did not take any medication. However, one hour before hospitalization, he suffered from severe retrosternal pain with nausea, sweating, shortness of breath, lethargy, and mild weakness, which increased in severity. The patient was alert during the physical exam with stable vital signs and mild cold sweating, blood pressure (BP)=110/70, pulse rate (PR)=60 beats per minute (BPM), respiratory rate (RR)=15/M, oxygen saturation level 98%; other physical exams were unremarkable.

According to dual type, ECG changes as the patient received ASA 300 mg, Plavix 300 mg, Pearl TNG, and Atorvastatin 80 mg in the emergency room. Transthoracic echocardiography revealed that the inferior wall had mild hyperkinesia and mild right ventricle systolic dysfunction.

**Figure 4.**
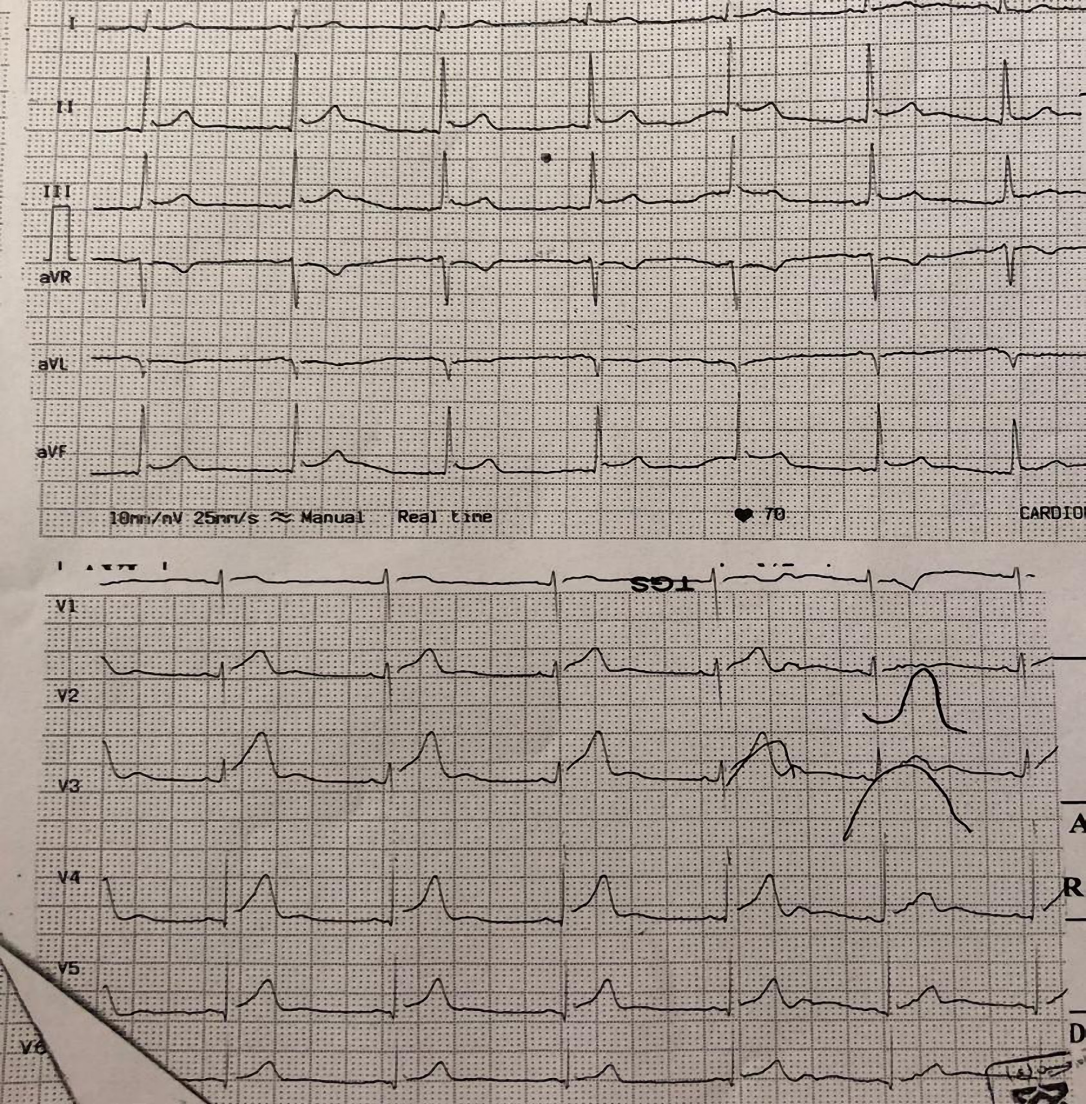
The significant return of the patient's ECG ischemic changes in inferior leads with primary treatment. Pericarditis changes have remained.

The patient was transferred to CCU with a diagnosis of infero postero-RV MI. In CCU, the patient received anti-ischemic therapy, including Trinitroglycerin (TNG) infusion with the minimum dose and recurrent BP check. Retrosternal chest pain relatively subsided and returned ST-elevation near baseline in II, III, AFV, RV4 (Figure 4).

**Figure 5.**
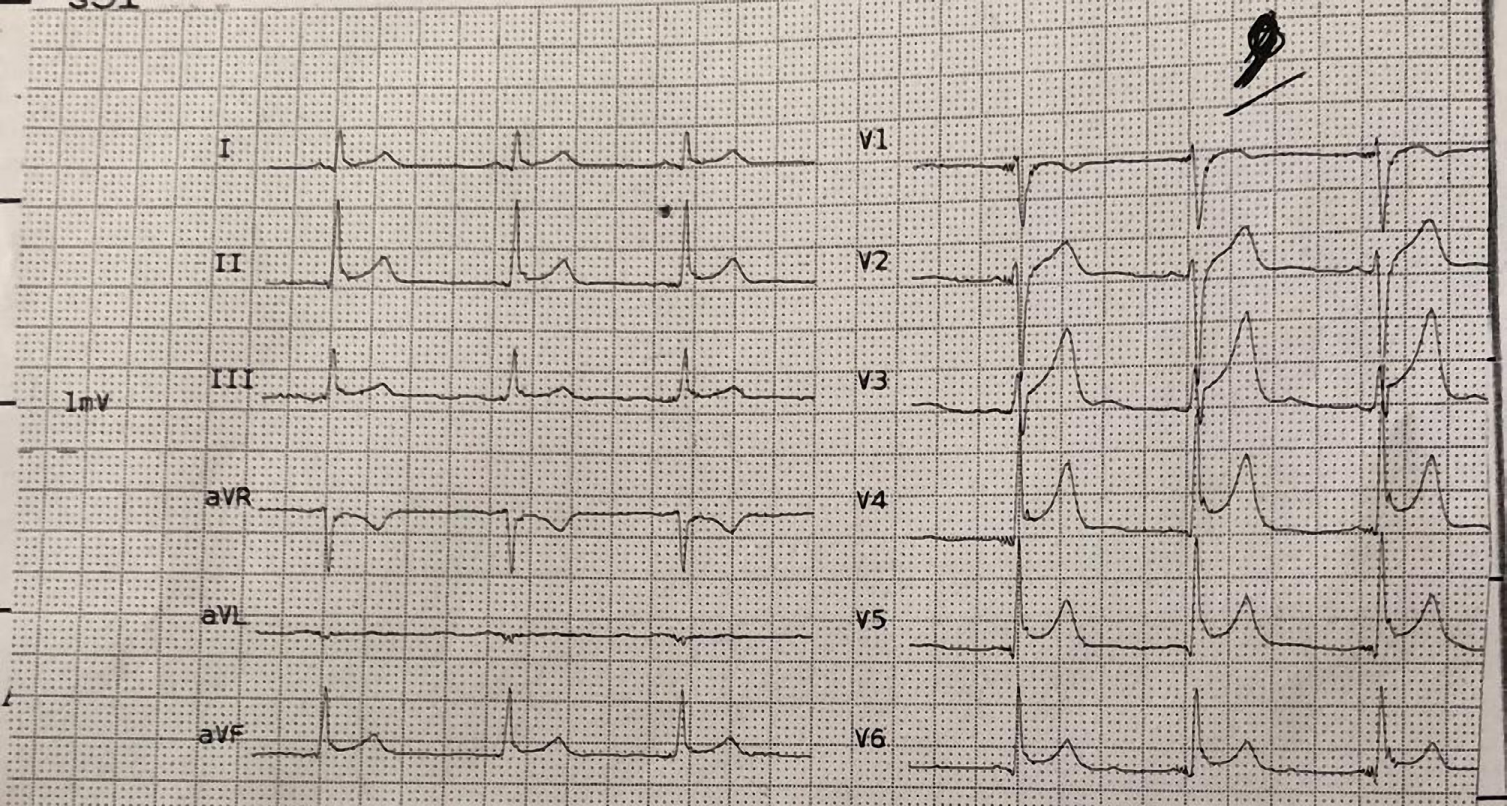
The complete return of ischemic changes and remaining changes related to pericarditis following vasodilator.

Due to good blood pressure, low age, and good performance of the right ventricle (RV) in TTE, TNG infusion continued. The first qualified troponin I was negative. 20 minutes later, ECG returned all ischemic changes to baseline, but generalized ST-elevation remained in favor of pericarditis (Figure 5).

At the same time, the patient’s retrosternal pain completely disappeared and the pleuritic pain, although not eliminated, decreased in severity. The patient’s second and third troponin was also negative.

**Figure 6.**
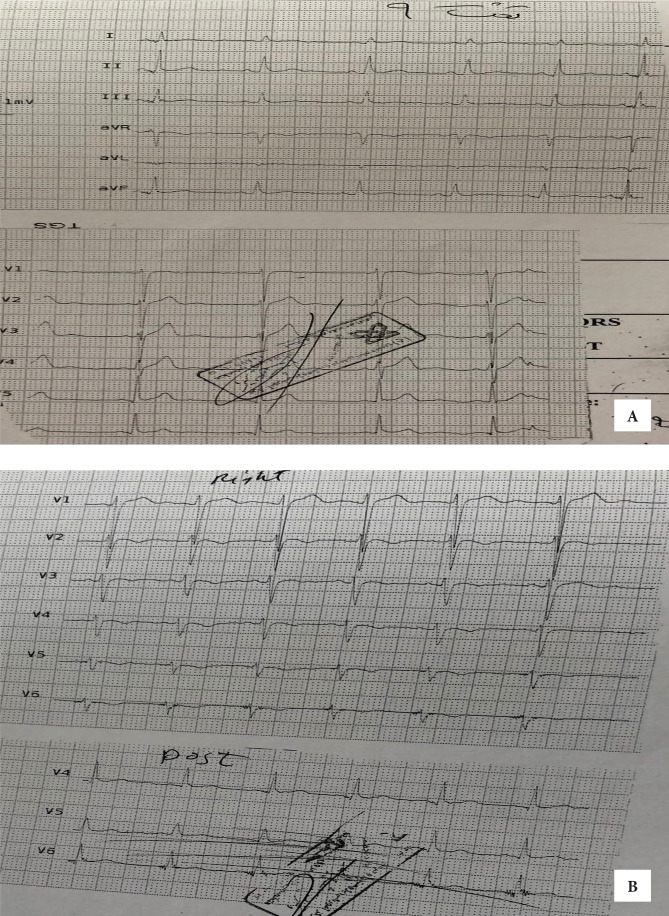
Patient's electrocardiogram during the third hospitalization day before coronary angiography; A – Left electrocardiogram, B – Posterior and right ventricle leads.

Creatine phosphokinase (CPK), creatine phosphokinase myocardial band (CPK MB), C-Reactive protein (CRP), erythrocyte sedimentation rate (ESR) were performed, all of which were in the normal range. No arrhythmias or recurrent pain recurred during the four-day hospitalization (Figure 6).

**Figure 7.**
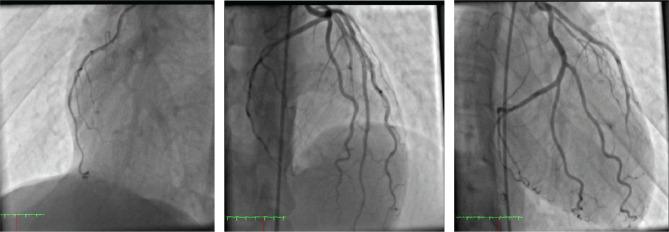
Coronary angiography images that show the patient's complete normal coronary arteries.

The complete disappearance of ischemic symptoms and partial reversal of pericarditis changes on ECG are evident. On the third day, he underwent coronary angiography, which revealed that the epicardial arteries were open and not narrow and without stenosis (Figure 7).

**Figure 8.**
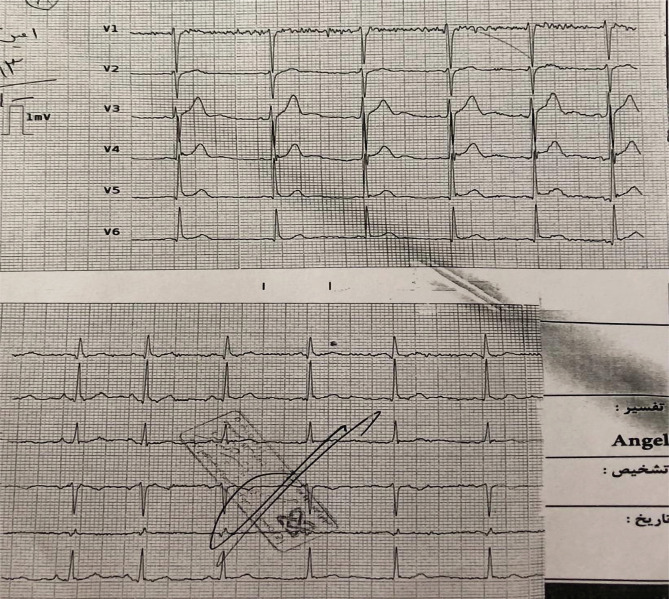
The patient's electrocardiogram on the fourth day after coronary angiography shows a complete reversal of all changes related to ischemia and pericarditis.

Transthoracic echocardiography (TTE) demonstrated normal ejection fraction without structural heart abnormality. He was discharged in good general condition on the fourth day and treated with ibuprofen, colchicine, and diltiazem. Two weeks later, all ECG changes returned, and ECG was near normal, with no new arrhythmias or changes (Figure 8).

Our patient had no new problems within three months after recovery and treatment with a calcium blocker during the next follow-up.

## Discussion

Prinzmetal angina or variant is mainly a vasospastic angina that was first described in 1959 by Prinzmetal M [7], an important heart functional disorder that can lead to ischemia and acute myocardial infarction type 2 [4]. This type of angina diagnosis is considered in those who have typical chest pain when significant stenosis in the coronary arteries cannot be found. This condition can be identified using coronary spasm-provoking tests. Microvascular dysfunction and other disorders are other causes of this condition [8, 9]. Although this condition can occur in a normal person without any trigger [10], several factors such as the use of amphetamines, cocaine, marijuana, alcohol, cigarettes, chemotherapy drugs and some antibiotics, antidepressants, anti-migraines, severe mental stress, the release of histamine for any reason, hypothermia and exposure to very cold weather, hyperventilation and inflammation of the coronary wall can trigger it [11]. It is more common in women than in men [12]. This difference in prevalence can lead to underestimating the actual causes in women [12]. The disease occurs with variable chest pain in the morning, which starts at rest and usually resolves spontaneously [13]. Vasodilators include nitrate and calcium blockers, common treatments [13]. The diagnostic criteria for vasospastic angina have been explained by the Coronary Vasomotion Disorders International Study Group (COVADIS) [8]. Pericardo myocarditis usually presents with diffuse changes in the ST and T segments that may be associated with cardiac enzymes [14]. Our patient was hospitalized and treated one month before admission with a diagnosis of acute pericarditis. During that hospitalization, the following finding confirmed the correct diagnosis of pericarditis: extensive ST-segment elevation with a pericarditis pattern accompanied by PR-segment depression, along with normal trans-thoracic echocardiography, an appropriate response to treatment with NSAID and resolved ECG changes in later follow-up. Negative cardiac enzymes and the absence of tachycardia at rest also confirmed the absence of myocardial involvement in that hospitalization. The patient also had an asymptomatic 20-day period. On the other hand, in the new current stage of the disease, the patient had similar pleural pain 24 hours before hospitalization, similar to the previous hospitalization. But the reason the patient went to the hospital this time was retrosternal pain with a heavy chest, which was accompanied by an upward convexity ST-segment elevation in infero septo-RV areas which were associated with inferior wall hypokinesia and right ventricle systolic dysfunction in concomitant transthoracic echocardiography. All of these confirm the presence of an obstruction in the coronary artery nutrient of these areas of the myocardium. Receiving a vasodilator caused a complete subside of retrosternal compressive pain, disappearance of ischemic changes in ST-segment, persistence of pleural pain, and generalized changes in ST-segment. The observed pericardial changes remained in the inferior leads after the removal of the ischemic changes. The result of angiography with full patent coronary arteries lacks premature atherosclerosis evidence, which suggests causes other than atherosclerotic occlusion of the coronary arteries for this patient, of which pericardial inflammation is the most likely.

## Conclusion

Pericarditis is a common cardiac disease that can lead to ischemic or even myocardial infarction clinical and paraclinical symptoms by triggering spasms in the coronary arteries. In this kind of coronary spasm, if there is no spontaneous reversal and timely intervention, the potential for myocardial infarction, serious myocardial damage, and even sudden cardiac death may occur.

## Acknowledgments

### Conflict of interest

The authors confirm that there are no conflicts of interest.

### Consent for publication

Patient consent was taken prior to writing this case report.

### Authorship

HS gathered the required data and reviewed the literature. MJM interpreted the laboratory findings and revised the final article. Both authors participated in writing the manuscript and read and approved the manuscript.
